# Comparison of PlanetScope, Sentinel-2, and landsat 8 data in soybean yield estimation within-field variability with random forest regression

**DOI:** 10.1016/j.heliyon.2023.e17432

**Published:** 2023-06-19

**Authors:** Khilola Amankulova, Nizom Farmonov, Parvina Akramova, Ikrom Tursunov, László Mucsi

**Affiliations:** a Department of Geoinformatics, Physical and Environmental Geography, University of Szeged, Egyetem Utca 2, Szeged 6722, Hungary; b Department of Hydrology and Ecology, “TIIAME” NRU Bukhara Institute of Natural Resources Management, Gazli Avenue 32, Bukhara, Uzbekistan

**Keywords:** Soybean yield, Remote sensing, PlanetScope, Sentinel-2, Landsat 8, Random forest

## Abstract

Accurate timely and early-season crop yield estimation within the field variability is important for precision farming and sustainable management applications. Therefore, the ability to estimate the within-field variability of grain yield is crucial for ensuring food security worldwide, especially under climate change. Several Earth observation systems have thus been developed to monitor crops and predict yields. Despite this, new research is required to combine multiplatform data integration, advancements in satellite technologies, data processing, and the application of this discipline to agricultural practices. This study provides further developments in soybean yield estimation by comparing multisource satellite data from PlanetScope (PS), Sentinel-2 (S2), and Landsat 8 (L8) and introducing topographic and meteorological variables. Herein, a new method of combining soybean yield, global positioning systems, harvester data, climate, topographic variables, and remote sensing images has been demonstrated. Soybean yield shape points were obtained from a combine-harvester-installed GPS and yield monitoring system from seven fields over the 2021 season. The yield estimation models were trained and validated using random forest, and four vegetation indices were tested. The result showed that soybean yield can be accurately predicted at 3-, 10-, and 30-m resolutions with mean absolute error (MAE) value of 0.091 t/ha for PS, 0.118 t/ha for S2, and 0.120 t/ha for L8 data (root mean square error (RMSE) of 0.111, 0.076). The combination of the environmental data with the original bands provided further improvements and an accurate yield estimation model within the soybean yield variability with MAE of 0.082 t/ha for PS, 0.097 t/ha for S2, and 0.109 t/ha for L8 (RMSE of 0.094, 0.069, and 0.108 t/ha). The results showed that the optimal date to predict the soybean yield within the field scale was approximately 60 or 70 days before harvesting periods during the beginning bloom stage. The developed model can be applied for other crops and locations when suitable training yield data, which are critical for precision farming, are available.

## 1 Introduction

Today, the most important agricultural indicators is crop productivity [1]. Accurately predicting crop yields in near real-time at the plot or farm scale is crucial [2] for generating early warning information, identifying low-yield zones, and performing site-specific management to prevent potential yield losses in the context of climate change and population growth,. Yield forecasting has direct implications for farmers’ incomes, food security policies, import–export policies, and food storage [3].

Soybean is among the most important source of protein for people all over the world and is a high-quality feed for animals [4]. It is estimated that one-third of annual and oilseed crops are covered by soybeans, according to the forecasts of the European Commission. Because of the strong demand for food by 2030, the production of soybean products is expected to continue to grow (EU Agricultural Outlook, accessed on April 17, 2020). While determining the growth stage where potential yield is affected, management activities toward increasing soybean yield output are most effective. For instance, the growth stage at which fertilization, frost or hail, moisture stress, plant diseases, and pesticide application occur, the yield will be affected. The vegetative (V) and reproductive (R) phases of crop development are distinguished by the system of soybean growth periods. Crop phenology can be estimated using satellite VI time-series signature (e.g., normalized difference vegetation index (NDVI)). This can be done simply by the extraction of crop-specific temporal metrics related to crop phenology (e.g., maximum NDVI).

Remote sensing (RS) has been a key focus in monitoring the growth of crops and predicting yields during the growing season using spectral bands and vegetation indices (VIs) [5]. The introduction of GPS, the Internet of Things, Earth observation (EO), and machine learning (ML) techniques in agriculture assist farmers in obtaining real-time information about their fields. In this regard, several EO-free and commercial satellites have been launched over the past decades. For instance, the Landsat 8 (L8) OLI long-term historical datasets provide excellent opportunities for the assessment, forecasting, and development of agricultural productivity models and maps at the field and country levels [6]. L8 complements the more than four million scenes captured by previous Landsat missions that are freely available on the Internet [7]. Meanwhile, newly developed EO systems that offer increased spatiotemporal resolutions (e.g., Sentinel-2 [S2] and PlanetScope [PS]) enable advanced agricultural studies. PS is a constellation of nanosatellites (Doves) provided by Planet that collects very high spatial resolution imagery [8], whereas CubeSats provide daily imagery covering 200 million km^2^/day. The PS constellation of 130 satellites is the most likely to obtain cloud-free images for crop forecasting and imaging of the entire Earth's surface with about 3-m spatial resolution [9]. This constellation of PS has been used for real-time forest monitoring, plant growth phenology, and crop yield prediction [10]. Meanwhile, S2 carries the twin MultiSpectral Instrument (MSI) satellites A + B onboard as part of the Copernicus program of the European Space Agency's enhanced precision agriculture applications [11]. S2 images the Earth's surface in 13 spectral bands ranging from visible to shortwave infrared. In this respect [12,13], achieved successful results using the S2 imagery to yield estimation in their research.

The electromagnetic spectrum's visible red, green, and blue bands and near-infrared (NIR) bands have been widely used for monitoring crop cover, crop health, soil moisture, nitrogen stress, and crop yields [14,[14], [14], [15], [16], [17], [18]]. When evaluating larger and spatiotemporal datasets, more advanced data analysis algorithms have also gained popularity along with the rise in computational processing capabilities [19]. With the help of remotely sensed VIs, ML techniques, including random forest (RF) and neural networks, have consistently been used to forecast crop productivity [[20], [21], [22], [23], [24]]. For instance, Schwalbert et al. [19] performed a satellite-based soybean yield estimation by combining ML and weather data in southern Brazil. They used satellite-derived NDVI, enhanced vegetation index, land surface temperature, and precipitation as input parameters for the yield prediction model. In their research, long short-term memory gave better results with a MAE of 0.42 Mg ha^−1^–70 days before the harvesting phase. Meanwhile, Pejak et al. [25] conducted soya yield prediction at the field level based on S2 imagery and soil variables with ML algorithms in Upper Austria. They used crop yield data provided by a yield monitoring system onboard a combine harvester as ground-truth data. In this previous study, a new approach (polygon–pixel interpolation) was developed to fit the yield data with satellite images. As a result, stochastic gradient descent (SGD) regression performed accurate yield estimation with an MAE of 0.436 t/ha and an *R*-value of 0.83%. In another study, Andrade et al. [26] investigated soybean yield prediction using RS and crop yield at the field scale. Multiple linear regression models were developed at the soybean growth stages based on L8 and S2 NDVI. They found that soybean grain yield can be predicted 29 and 46 days after planting, with a mean error of predictions of 153.9 kg/ha. Previous studies support the individual capability of S2 and L8 for soybean yield estimation. However, the potential of these sensors has not been fully explored yet. However, the feasibility of estimating within-field soybean yield variability has not been fully explored, and there is a need for integrating multiplatform data and data automation. Advances in satellite imagery collection have led to finer spatial resolution (up to 1 m) and more frequent observations (nearly daily observations), thereby enabling the collection of more information at field and within-field scales to support agricultural operations. Most of these studies relied on only RS data, which limited their applicability in other areas. EO-based studies on mapping yields at high resolution often lack high-resolution yield data for training and validation. The accuracy of grain yield models can be improved by combining RS data with GPS combine harvesters. Thus, further studies and developments are necessary to achieve a robust model for soybean yield prediction.

This study primarily aims to evaluate the capability of PS, S2, and L8 and their spatiotemporal coverage in soybean yield estimation within-field variability with an ML algorithm. To the best of our knowledge, this is the first case study to have used 8-band PS (PSB.SD) imagery and a combination of RS data with environmental data (e.g., climate and LiDAR digital terrain model [DTM]) in soybean yield estimation. RF models were trained and validated using yield data from a harvester machine.

This research contains four key questions developed to study how different combinations of data, in terms of both type and spatiotemporal resolution, influence the accuracy of soybean yield at the field level.

1. How do the spatial and temporal resolutions of PS, S2, and L8 affect the precision of yield prediction?
2. Does the calculation of additional VIs contribute extra information to the estimation model?
3. How does the estimation accuracy differ when S2, L8, and PS data are combined with environmental data?
4. Which stage of soybean growth and individual satellite data image offers the most accurate estimation?

## 2 Material and methods

### 2.1 Field sites

**Fig. 1:**
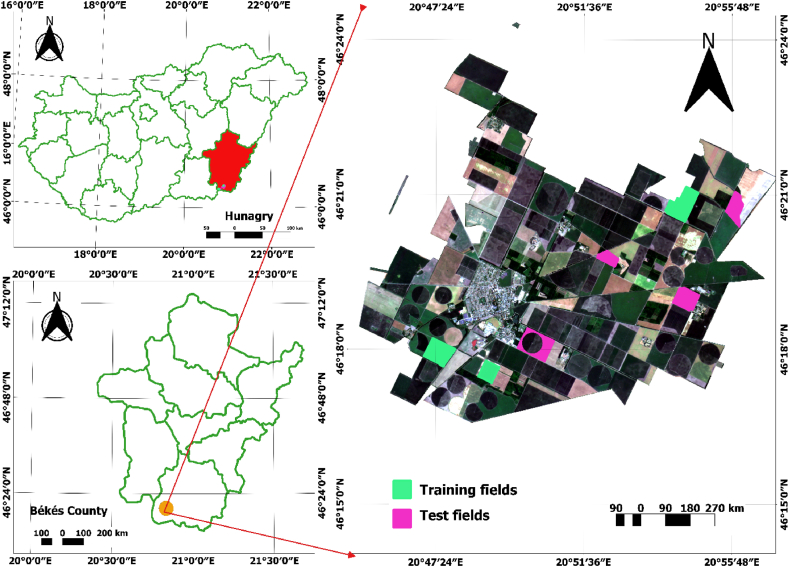
Study area (natural color composite from PlanetScope imagery; bands: RGB (4, 3, 2): acquisition date: June 28, 2021).

The study parcels are in Mezőhegyes town, Békés county, in southeast Hungary close to the Romanian border (latitude 46°19′N, longitude 20°49′E), where the Mezőhegyes experimental farm is situated (Fig. 1). The town has a population of 4950 and a total administrative area of 15,544 ha. A total of seven parcels were selected for analysis. Three fields were used for model development, and the remaining fields were used for validation processes. Soybean is the most cultivated crop type, which covers a 1090 ha area in total. The average field size is 36 ha, whereas the maximum area reaches 75 ha. Chernozem is a very popular kind of soil that fosters plant development and produces abundant crops. Because of their high levels of lime, meadow and lowland chernozem make a fantastic foundation for field plant production. High agricultural yields and great agronomic conditions are provided by the fertile soil of chernozem, which is best suited for growing crops, particularly cereals and oilseeds. The experimental farm of Mezőhegyes, Mezőhegyesi Ménesbirtok Zrt., has a significant impact on both Mezőhegyes and the nearby communities. The average annual rainfall was 645 mm (428.9 mm in crop) for 2021. The average annual temperatures in the study site range between 7.8 °C and 11.1 °C.

### 2.2 Soybean yield data

High-resolution soybean yield data were collected between the 7th of September and the 18th of October during the 2021 harvesting time using a combine machine equipped with a yield monitoring system and GPS. In Hungary, soybean yield crops are typically sowed in April and harvested in September. The raw yield data were cleaned to remove inaccurate grain yield measurements caused, for instance, by the combine harvester's harvesting dynamics and the precision of the positioning data [27]. When harvested rows overlap, commercial yield monitors are prone to producing inaccurate data, which would indicate a poor crop yield in particular sections of the field. Thus, straight-line sequences of locations with yields close to zero were eliminated. Cleaning inaccurate grain yield includes determining combine delay times and removing “overlapped” data, especially data from near-end rows. All GPS crop yield points obtained from the combine harvester were uploaded in the shapefile format in QGIS. In this format, the data are organized in attribute tables and hence are easier to process and filter. First, yield points with zero and near-zero values were deleted from the attribute table. Second, we selected homogenous yield points with the same distance and swath width as the header of the combine harvester, and the other yield points were excluded; this resulted in tractor lagging during harvesting. Finally, the edge of the parcel was cut to avoid mixed pixels. Data on crop yields were calibrated and filtered by the company that owns and runs the farming operations in the study area. Only data on crop yields that had the same width and length as the combine harvester's header dimensions (i.e., 2 m by 6 m) remained. We next transformed the crop yield data to raster format using QGIS v.3.16's inverse distance weighted interpolation method to 3-, 10-, and 30-m resolutions to match the resolution of the satellite images. To make a fair comparison, we performed interpolation corresponding to the spatial resolution of the PS, S2 and L8 for the calibration and pixel matching between crop yield data and satellite images.

### 2.3 RS data

#### 2.3.1 PS imagery and preprocessing

A total of 81 available cloud-free PS Level-3 Surface Reflectance products collected during the soybean growing phase between April and October were downloaded from the Planet Explorer website (https://www.planet.com/explorer/; accessed on August 25, 2022). In this study, a new generation of DOVE CubeSat, PS Super Dove (PSB.SD), was used. The PSB. SD instrument provides eight spectral bands (red edge, red, green, green I, yellow, blue, coastal blue, and NIR) with a pixel size of 3 m and near-daily global time revisit. The PS orthorectified product was geometrically and radiometrically corrected for surface reflection and projected to a UTM/WGS84 cartographic map projection (Planet Team, 2017). These images were harmonized with S2 for consistent radiometry. The first coastal blue band was discarded from this study, and images were subset to the area of interest (AOI). Finally, all PS bands were layer-stacked together to derive VIs and crop phenological stages.

#### 2.3.2 S2 image processing

During the study period, we downloaded 18 cloud-free S2 Level-2A (L2A) satellite images from the Copernicus Open Access Hub website (https://scihub.copernicus.eu/dhus/#/home; accessed on September 5, 2022). A Level-2A product provides images of the bottom of atmosphere reflectance covering the visible and NIR spectral range derived from associated Level-1C datasets. MSIs are equipped on S2 A and B, allowing agricultural monitoring on regional and global scales at various spatial resolutions (10, 20, and 60 m) [28]. A single S2 satellite can map the entire globe once in every 10 days, and the combined constellation revisit is 5 days. Band 1 (coastal aerosol), Band 9 (water vapor), and Band 10 (cirrus) were excluded and not considered in this research. The bands with resolutions of 20 and 60 m were downscaled to 10 m to ensure that all channels were concatenated with aligned pixels. Further, stacked datasets were clipped to AOI to calculate the VIs.

#### 2.3.3 Landsat 8

Because of their applications in agricultural studies, remotely sensed L8 OLI images are vital for this paper. The L8 OLI design is an advancement in Landsat sensor technology, allowing for the collection of a significantly greater number of images per day with improvements in signal-to-noise ratio, as well as spectral and radiometric resolutions [6]. Additionally, the Landsat archive and the data collected by L8 OLI, which has 30 m spatial and 16 days temporal resolutions, are free to download from the United States Geological Survey data center (https://earthexplorer.usgs.gov/; accessed on April 10, 2022) within 24 h of acquisition. Sixteen relatively cloud-free L8 OLI Level-2 Collection 2, Tier 1 scenes were ordered and downloaded from EarthExplorer Bulk Download Application. In this study, six spectral bands, four visible and NIR bands, and two shortwave infrared (SWIR) bands present in these images except Band 1 (ultra blue, coastal aerosol) were chosen during the growing season. These images were already atmospherically and geometrically corrected and orthorectified at this level.

#### 2.3.4 Vegetation indices

Based on prior yield estimation research, four widely used VIs [19,25,29] were calculated on ERDAS IMAGINE 2020 from PS, S2, and L8 images (Table 1). NDVI [30] and the green NDVI (GNDVI) [31] are well-established and can simply retrieve spectral reflectance indicators of crop heat stimuli. Gitelson et al. [31] developed the GNDVI to address saturation issues observed with NDVI for some vegetation types at later growth stages. Because GNDVI uses the green band as an alternative to the red band in the NDVI estimator, it is presumed to be more useful for assessing leaf chlorophyll variability when the leaf area index (LAI) is relatively higher [31]. Gianelle et al. [32] acknowledged that GNDVI was less influenced by saturation and thus yielded consistent results of various vegetation effectiveness leading indicators. Meanwhile, the soil adjusted vegetation index (SAVI) includes a soil adjustment factor to make up for the difference in the influence of the soil's brightness. According to the amount of visible soil, this factor can range from 0 to 1. Maximum levels should be used in areas where there is more visible bare soil [33]. Although MTVI2 and MTVI are almost identical, MTVI2 is regarded to be a superior indicator of green LAI. It accounts for soil background signatures while retaining sensitivity to LAI and resistance to chlorophyll influence [34].

**Table 1:** Multispectral VIs investigated in this study.

Index	Equation	Reference
Normalized difference vegetation index (NDVI)	$\frac{NIR-Red}{NIR+Red}$	[35]
Green normalized difference vegetation index (GNDVI)	$\frac{NIR-Green}{NIR+Green}$	[31]
Soil adjusted vegetation index (SAVI)	$\left(1+L\right)\frac{\left(NIR-Red\right)}{\left(NIR+Red+L\right)}$	[36]
Modified triangular vegetation index (MTVI2)	$\frac{1.5\left[1.2\left(NIR-Green\right)-2.5\left(Red-Green\right)\right]}{\sqrt{{\left(2NIR+1\right)}^{2}-\left(6NIR-5\sqrt{Red}\right)}-0.5}$	[34]

In SAVI, the “L” value was set to 0.5, and the soil line and slope were defined according to the soil reflectance relationship between B3 and B4.

#### 2.3.5 Monitoring of soybean phenology development

The growing season is a dynamic time for crop phenology [37]. Throughout the growing season, phenological observations and transition dates were noted for the seven soybean fields twice a month. Field measurements and spectral reflectance patterns derived from satellites were compared. NDVI, GNDVI, and SAVI were used to define phenological patterns, whereas MTVI2 was used to measure and assess leaf chlorophyll content at the canopy scale while being largely insensitive to the LAI. All satellite images were used to extract the time series of the VIs.

**Fig. 2:**
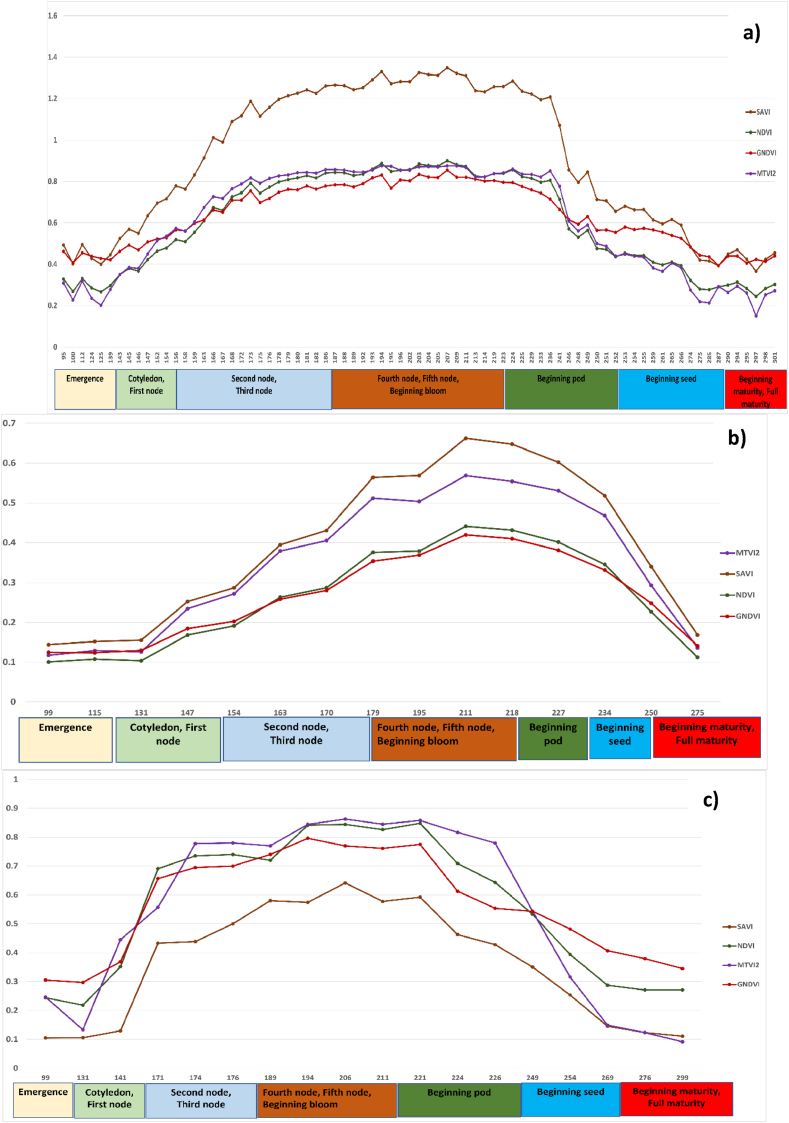
Soybean phenological stages based on (a) PlanetScope, (b) Sentinel-2 and (c) Landsat 8 VIs during the growing season.

The four VIs (NDVI, GNDVI, SAVI, and MTVI2) calculated using multitemporal PS, L8, and S2 were used to reflect the soybean growing stages covering the period from soybean planting to harvesting. Fig. 2 illustrates the different temporal patterns acquired from the RS-based monitoring of the soybean growing season. Points were obtained using random points inside the polygon tool in QGIS 3.16. The VI values were extracted on a point sampling tool in the seven fields using a free and open-source plugin in QGIS to determine the crop phenology and transition dates. The 65 points that were created randomly from each VI were then averaged and distributed over the stages of soybean development. The crop ages in the satellite images were calculated according to the day of the year (DOY).

### 2.4 Environmental data

#### 2.4.1 Precipitation and temperature

Monthly (1/24°, ∼4 km) gridded TerraClimate datasets for total precipitation (mm), maximum temperature (°C), and soil moisture (mm) were downloaded from the Google Earth Engine cloud platform [38]. TerraClimate incorporates a monthly climate and climatic water balance covering global terrestrial surfaces from the University of California Merced and various high and coarser-spatial-resolution climatological datasets (e.g., WorldClim and Japanese 55-year Reanalysis). Monthly accumulated datasets were obtained from April to October 2021. When compared with other climate datasets, these have a relatively high spatial resolution. As a result of the spatial distribution, we were able to detect spatial variations in rainfall and temperature across the study area. Finally, these datasets were fed into the yield prediction model as an input feature.

#### 2.4.2 Topographic variables

A 5-cm spatial resolution of a very accurate LiDAR DTM was obtained over the study area. The DTM data were acquired on the basis of airborne radar data collected on April 19, 2019. These data were resampled to 3-, 10-, and 30-m resolutions to match the spatial resolution of PS, S2, and L8 using the cubic convolution method in ERDAS IMAGINE 2020 software. This method was employed because the mean and standard deviation of the output pixels generally matched the mean and standard deviation of the input pixels more closely than any other resampling method even with the high computational costs. Rescaled datasets are used to calculate secondary variables, slopes, and aspects as input parameters for estimation models.

### 2.5 RF regression

RF regression (RF) is based on the decision tree algorithm and has been used to predict crop yield [39]. The RF model builds up tree predictors associated with different random vector values sampled independently. An RF model constructs decor-related decision trees during the training phase, and the overall model output is obtained by averaging the output values of all the individual trees. In the RF model, the learner bagging algorithm is used to train any single tree [40]. The performance of RF combines predictions from multiple ML algorithms to make a more accurate assessment than that of a single model, which is the main benefit of this approach over decision trees [41]. The RF ML technique was chosen in this research because previous studies have proven the effectiveness and superiority of this method over other algorithms (e.g., support vector, boosting regression, and multilinear regression) [42,43].

The “randomForest” package in R software was used to implement an RF model (Liaw et al., 2002). The number of trees produced in the regression forest (i.e., ntree) was set at 500, and the number of distinct predictors sampled at each node (i.e., mtry) was set to a default of the number of predictors (203) divided by 3. These two parameters were changed to optimize the RF model. Every time an RF model was developed, 70% of the dataset was utilized to train the models, and 30% of the dataset, which contained four fields not used in training, was used for validation. Using the layer combinations shown in Table 2, we examined how different combinations of data and different temporal coverages affect the estimation accuracy. First, the peak vegetative period as crop maximum growth was selected following phenological stages (V4–V5–R1) to train the model in the RF analysis. VI pixel values reached a peak period for all three satellites in July (187 and 223 DOY). Therefore, this month was chosen as the baseline to build the training model and test the yield prediction using spectral bands and VIs of each sensor from all available images acquired in July.

**Table 2:** Data integrations were examined in this study using RF.

Integration	Data layers
**Question 1: Sensor comparison**	
PS	PlanetScope bands
S2	Sentinel-2 bands
L8	Landsat-8 bands
**Question 2: Testing VIs individually and in combination with spectral bands of PS, S2 and L8**	
VI	VIs extracted from PS, S2 and L8
*P*S-VI	PlanetScope + VIs
S2-VI	Sentinel-2+VIs
L8-VI	Landsat-8+VIs
**Question 3 Combination of the Topographic and climate data to the best-performed integrated Spectral bands and VIs**	
*P*S-VI - Topographic	PlanetScope + VIs + DTM, Aspect, Slope
*P*S-VI - Topographic-Climate	PlanetScope + VIs + DTM + Aspect + Slope + Precipitation + Temperature
S2-VI - Topographic	Sentinel-2+VIs + DTM, Aspect, Slope
S2-VI - Topographic-Climate	Sentinel-2+VIs + DTM + Aspect + Slope + Precipitation + Temperature
L8-VI – Topographic	Landsat 8 + VIs + DTM, Aspect, Slope
L8-VI – Topographic - Climate	Landsat 8 + VIs + DTM + Aspect + Slope + Precipitation + Temperature
**Question 4: Identification of best performed single date image and growing stage**	
PS	PlanetScope image (July)
S2	Sentinel-2 image (July)
L8	Landsat 8 image (July)

The predicted yield data from test sites were compared with the observed yield from the harvester machine, and residuals were calculated. We calculated metrics, such as the coefficient of determination (*R*^2^), RMSE, normalized root mean squared error (NRMSE) and mean absolute error (MAE), to evaluate the accuracy of the prediction model using the following equations (1), (2), (3), (4).

(1) $$R^{2}=1-\frac{RSS}{TSS}$$

(2) $$RMSE=\sqrt{\frac{{\sum_{i=1}^{n}\left(y_{i}-{\hat{y}}_{i}\right)}^{2}}{n}}$$

(3) $$NRMSE=\frac{\sqrt{\frac{{\sum_{i=1}^{n}\left(x_{i}-y_{i}\right)}^{2}}{n}}}{y_{\max}-y_{\min}}$$

(4) $$MAE=\sqrt{\frac{\sum_{i=1}^{n}\left|x_{i}-y_{i}\right|}{n}}$$

## 3 Results

### 3.1 Phenology and date

VIs derived from the three sensors PS, S2, and L8 during the growing season demonstrated nearly identical and consistent temporal patterns as the VIs values based on plant spectral reflectance (NDVI, GNDVI, SAVI, and MTVI2) did. All VI values showed the lowest record at the beginning of the vegetative period. The VIs began to steadily increase after a few weeks (125–156 DOY), which denoted the initiation of the vegetative stages (e.g., the emergence of cotyledons) and significant soybean growth. The soybeans' growth reached its peak between 187 and 223 DOY, which is linked to the VIs’ highest values (Fig. 2). The soybeans entered the beginning pod and seed when the VIs started to decline at 224–260 DOY. At 261–301 DOY, the period of harvest and when the soybeans started to fully mature, the VIs recorded their lowest values.

### 3.2 Crop yield estimation with RF

**Fig. 3:**
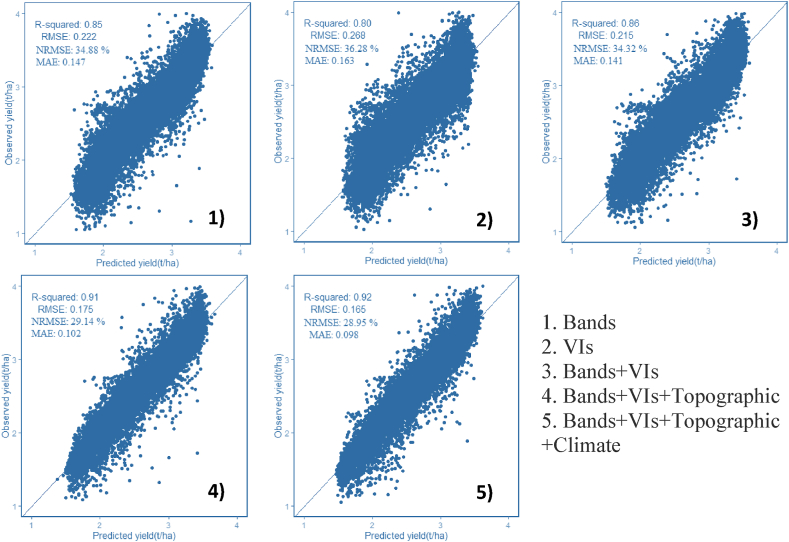
Scatter plots between the observed and predicted yields for the training data set using PS and combination of different explanatory variables (1, 2, 3, 4 and 5).

**Fig. 4:**
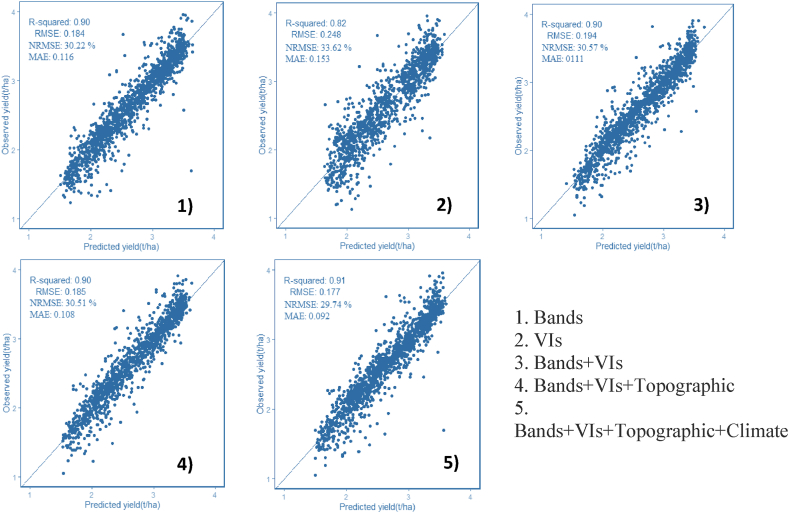
Scatter plots between the observed and predicted yields for the training data set using S2 and combination of different explanatory variables (1, 2, 3, 4 and 5).

**Fig. 5:**
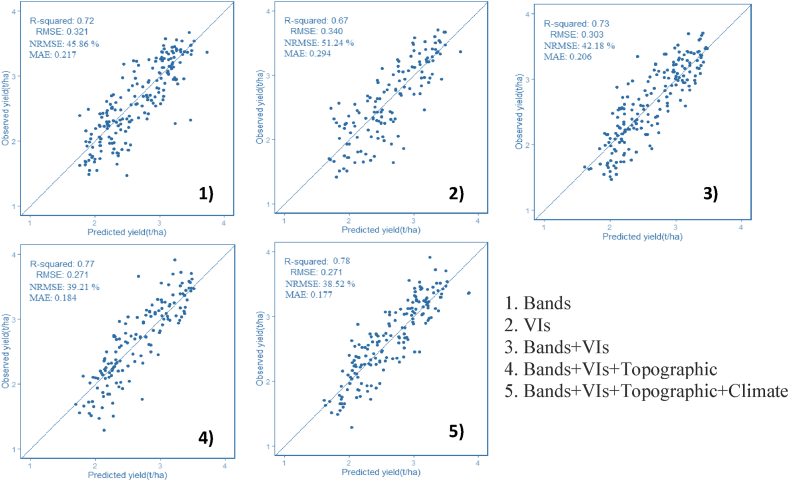
Scatter plots between the observed and predicted yields for the training data set using L8 and combination of different explanatory variables (1, 2, 3, 4 and 5).

The outcome of the regression analysis is displayed in Table 3, Table 4, Table 5. The results indicate that the use of the Fourth node, Fifth node, and beginning bloom dates coupled with RF regression and the 3-, 10-, and 30-m resolutions of PS (Fig. 3), S2 (Fig. 4), and L8 (Fig. 5) multispectral bands had the best performance with R2 and RMSE values ranging from 0.7 to 0.9 and 0.183 to 0.321 t/ha, respectively. Accordingly, the NRMSE coefficient ranges from 29.08% to 52.39%, and the MAE values range from 0.042 to 0.127 t/ha. Similarly, the VIs obtained from the three sensors for the same precise circumstance (R2 ranged from 0.63 to 0.82, RMSE found from 0.248 to 0.356 t/ha, MAE values obtained from 0.098 to 0.214 t/ha, while the NRMSE ranged from 40.93 to 55.05%) also worked reasonably well. The accuracy of the model trend also observed an increase as the vegetation period reached its peak at the end of July. Therefore, with all the data feeding methodologies here evaluated (VIs and 3-, 10-, and 30-m PS, S2, and L8 bands alone) with bands arguably the most accurate, within-field soybean yield variability may be calculated relatively correctly. The best-fitted dates were further selected (July 30 and 31) to combine environmental data (e.g., climate and topographic variables) to increase the model accuracy. All additional models developed in this study demonstrated enhanced yield estimation accuracy when compared with these spectral bands and VIs (Fig. 3, Fig. 4, Fig. 5).

**Table 3:** Root mean square error (RMSE) and coefficient of determination (*R*^2^) values were computed from the training dataset for RF using the July-derived vegetation indices (VIs) and spectral bands of PS.

PlanetScope	Bands				Indices			
Days	RMSE	R^2^	NRMSE %	MAE	RMSE	R^2^	NRMSE %	MAE
1-July	0.285	0.76	51.28	0.110	0.349	0.64	54.66	0.214
5-July	0.262	0.80	39.45	0.091	0.329	0.68	53.24	0.179
6-July	0.268	0.79	41.56	0.121	0.324	0.69	54.21	0.187
7-July	0.261	0.80	38.90	0.102	0.324	0.69	54.98	0.194
11-July	0.259	0.80	38.87	0.103	0.344	0.65	55.34	0.201
12-July	0.253	0.81	38.57	0.093	0.340	0.66	55.05	0.147
13-July	0.248	0.82	38.21	0.082	0.321	0.70	53.12	0.139
14-July	0.254	0.81	38.86	0.089	0.322	0.70	52.67	0.134
22-July	0.231	0.84	34.36	0.078	0.353	0.64	54.38	0.206
23-July	0.217	0.86	32.45	0.069	0.325	0.69	53.89	0.187
24-July	0.230	0.84	33.67	0.087	0.329	0.68	54.83	0.185
25-July	0.235	0.83	33.98	0.090	0.335	0.67	54.90	0.198
27-July	0.205	0.87	30.89	0.067	0.356	0.63	55.87	0.213
29-July	0.227	0.85	34.83	0.074	0.313	0.71	52.86	0.145
**31-July**	**0.222**	**0.85**	**33.58**	**0.083**	**0.268**	**0.80**	**48.62**	**0.098**

The highest value is in bold according to the best fit to *R*^2^ and the corresponding RMSE.

**Table 4:** RMSE and *R*^2^ values computed from the training dataset for RFRs using July-derived VIs and spectral bands of S2.

Sentinel 2	Bands				Indices			
Days	RMSE	R^2^	NRMSE %	MAE	RMSE	R^2^	NRMSE %	MAE
8-July	0.184	0.90	29.38	0.054	0.282	0.77	46.87	0.147
13-July	0.186	0.89	29.96	0.061	0.286	0.76	46.91	0.135
25-July	0.183	0.90	29.13	0.047	0.258	0.80	42.51	0.126
**30-July**	**0.184**	**0.90**	**29.08**	**0.042**	**0.248**	**0.82**	**40.93**	**0.119**

The highest value is in bold according to the best fit to R2 and the corresponding RMSE.

**Table 5:** RMSE and *R*^2^ values were evaluated from the training dataset for RFRs using July-derived VIs and spectral bands of L8.

Landsat 8	Bands				Indices			
Days	RMSE	R^2^	NRMSE %	MAE	RMSE	R^2^	NRMSE %	MAE
14-July	0.314	0.70	52.39	0.138	0.338	0.66	52.93	0.144
**30-July**	**0.321**	**0.72**	**50.24**	**0.127**	**0.340**	**0.67**	**52.04**	**0.135**

The highest value is in bold according to the best fit to R2 and the corresponding RMSE.

The end of July was the peak vegetative period and thus gave accurate yield estimation results for each sensor's spectral bands and VIs. Developed models for all images in July and July 31 for PS and July 30 for S2 and L8 were chosen to combine with environmental data. First, integrated bands and VIs were used for the regression analysis. Then, environmental data were combined with the bands and VI models. The root means square error decreased and the R^2^ tended to increase. The highest and most accurate estimation models were observed when all of the datasets were combined in the case of the three sensors. Fig. 3, Fig. 4, Fig. 5 represent the combination of the data layers used in the RF analysis.

When the three constellations combined with environmental data were compared using RF, PS had the most accurate result with an RMSE of 0.165 kg/ha, followed by S2 and L8 with RMSE values of 0.177 and 0.271 kg/ha, respectively. Fig. 3, Fig. 4, Fig. 5 show how accuracy metrics changed when all datasets were integrated. The most accurate estimated training model that came from the combined Bands–VIs–Topographic–Climate–RF was used to test and validate the efficiency of the model on independent datasets.

**Fig. 6:**
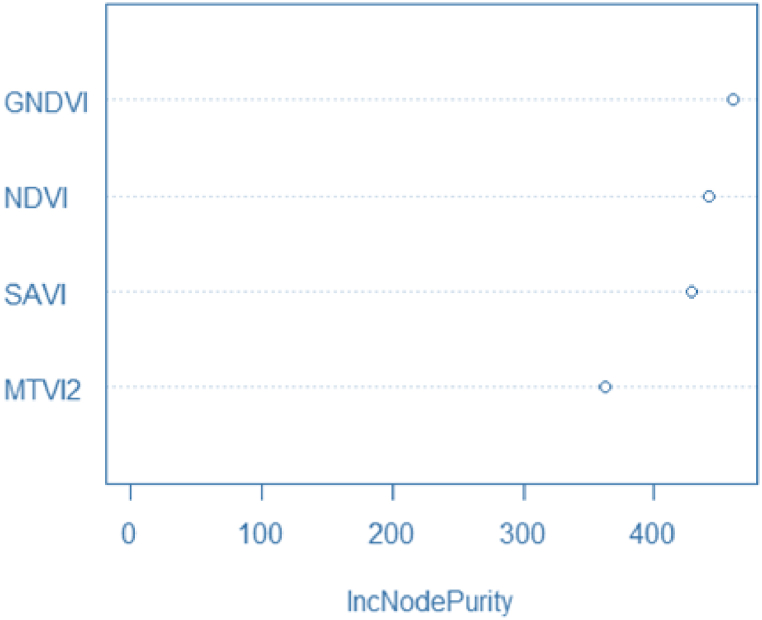
Example of variable importance (IncNodePurity values) list of the VIs random forest model.

The S2 VI-based model had slightly higher accuracy than the PS VI-based model with RMSE values of 0.248 kg/ha and 0.268 kg/ha, respectively, for the training data (Fig. 3, Fig. 4, Fig. 5). The higher accuracy is attributed to the higher spectral and radiometric resolution of S2 imagery and the inclusion of more spectral bands (i.e., three red edge and SWIR bands). The VIs derived from L8 had the lowest accuracy with an RMSE = 0.340 kg/ha because of a decrease in the ability to capture within-field yields with moderate spatial resolution. As a part of the RF analysis, we also examined the variable importance of the RF model using all VIs (Fig. 6). We found that GNDVI and NDVI with an IncNodePurity score just below 500 are the most promising variables, followed by SAVI, for all PS, S2, and L8. Lastly, MTVI2 was the least important variable in the model.

### 3.3 Spatial prediction and validation

**Fig. 7:**
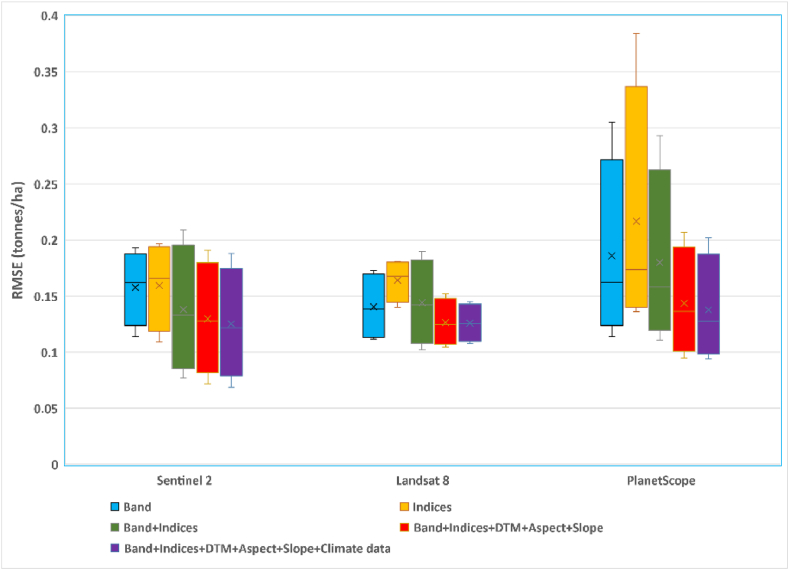
Box plots exhibiting the effect of the different combinations and sensors on RF models based on the validation dataset.

**Fig. 8:**
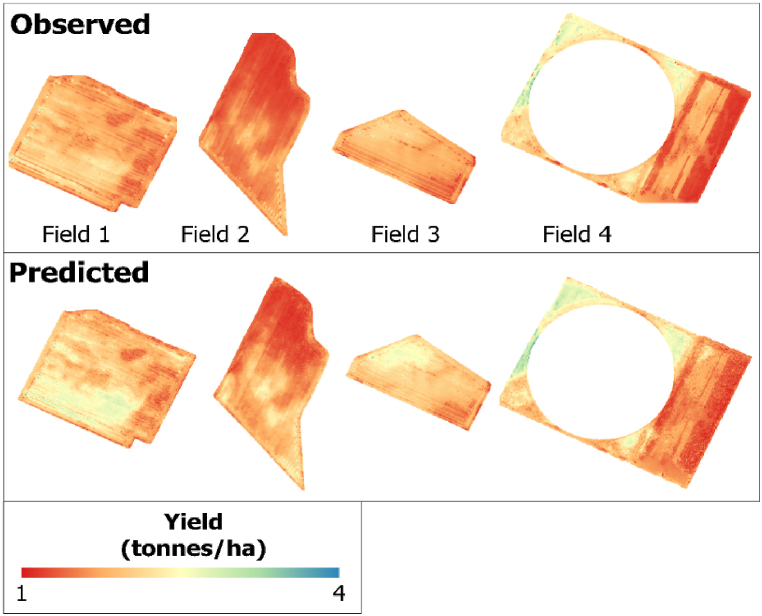
For a validation field, the observed yield was interpolated from the harvester machine data (upper), while the predicted yield was derived from the PS–VIs–Environmental RF model (bottom).

**Fig. 9:**
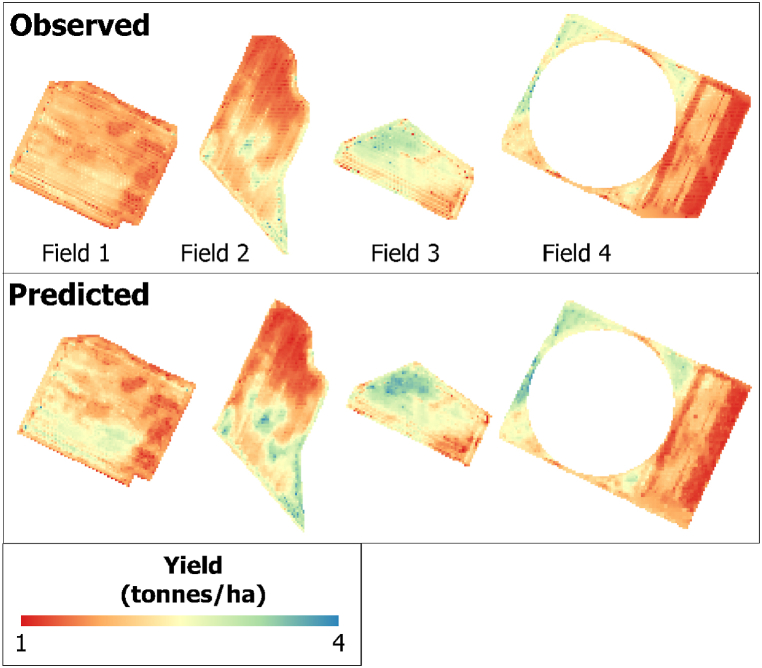
For the validation fields, the observed yield was interpolated from the harvester machine data (upper), while the predicted yield was derived from the S2–VIs–Environmental RF model (bottom).

**Fig. 10:**
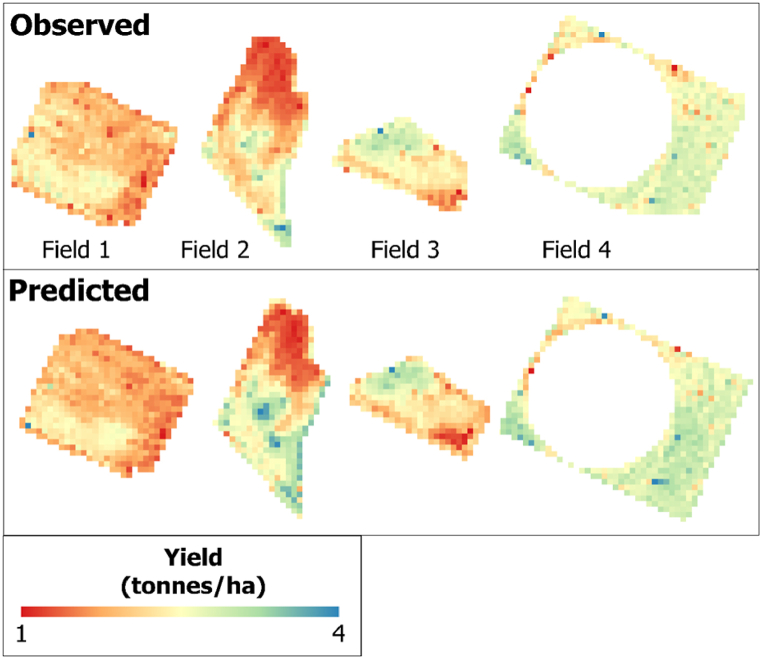
For a validation field, the observed yield was interpolated from the harvester machine data (upper), while the predicted yield was derived from the L8–VIs–Environmental RF model (bottom).

By applying the best-performing RF-based Bands + Vis + Topographic + Climate model that combined all datasets, we generated a crop yield spatial distribution map of the validation field for each pixel. The three satellite images captured during the peak season of the phenological stage were used for validation because they were found to be the best during the training model development. Fig. 8, Fig. 9, Fig. 10 show the observed and predicted soybean yields for individual validation parcels corresponding to each satellite sensor. In this study, actual crop yield data were recorded by the harvester machine equipped with GPS and a yield monitoring system. Observed soybean yields as cloud points were first filtered to remove incorrect values. Furthermore, point yield data were interpolated to 3-, 10-, and 30-m resolutions corresponding to the PS, S2, and L8 pixel sizes. We studied a total of four soybean fields used to validate the prediction model and evaluate model efficiency. We compared the predicted yield map result with the observed crop yield provided by the combine tractor equipped with a yield monitoring system. The soybean distribution map derived from RF visually reflected the general pattern of the observed yield, with relatively little variation in the within-field patterns. We also identified areas where the model underestimated and overestimated yields using the predicted yield map. Regardless of these trends, the model seems to produce reasonably accurate predictions of the within-field yield variability for specific fields, with RMSE values ranging from 0.069 to 0.202 t/ha. When comparing the satellites according to the results shown in Fig. 7 (Table 6, Table 7, Table 8), PS and S2 outperformed L8.

**Table 6:** RMSE and *R*^2^ values for the validation datasets using PS and environmental data.

Sensor type	Fields	Metrics	Bands	Vegetation Indices	Band + VIs	Bands + VIs + Topographic	Band + Indices + Topographic + Climate data
PlanetScope	Field 1	R^2^	0.51	0.52	0.55	0.70	0.70
		RMSE	0.153	0.152	0.145	0.119	0.112
		NRMSE %	49.98	50.65	50.25	51.15	49.90
		MAE	0.093	0.205	0.112	0.091	0.082
	Field 2	R^2^	0.74	0.63	0.74	0.82	0.82
		RMSE	0.114	0.136	0.111	0.095	0.094
		NRMSE %	54.21	57.11	52.01	44.02	48.20
		MAE	0.101	0.151	0.091	0.068	0.064
	Field 3	R^2^	0.63	0.53	0.64	0.72	0.74
		RMSE	0.172	0.195	0.174	0.154	0.143
		NRMSE %	62.80	68.02	58.80	49.60	48.40
		MAE	0.123	0.142	0.121	0.092	0.091
	Field 4	R^2^	0.69	0.50	0.71	0.86	0.87
		RMSE	0.305	0.384	0.293	0.207	0.202
		NRMSE %	38.92	48.54	38.02	36.82	36.01
		MAE	0.140	0.164	0.154	0.152	0.141

**Table 7:** RMSE and *R*^2^ values for the validation datasets using S2 and environmental data.

Sensor type	Fields	Metrics	Bands	Vegetation Indices	Band + VIs	Bands + VIs + Topographic	Band + Indices + Topographic + Climate data
Sentinel 2	Field 1	R^2^	0.73	0.53	0.71	0.72	0.73
		RMSE	0.114	0.147	0.111	0.110	0.109
		NRMSE %	54.78	55.67	54.34	54.10	52.28
		MAE	0.127	0.141	0.131	0.129	0.125
	Field 2	R^2^	0.89	0.75	0.88	0.89	0.90
		RMSE	0.171	0.109	0.077	0.072	0.069
		NRMSE %	28.66	30.64	27.12	28.70	28.30
		MAE	0.107	0.129	0.121	0.112	0.097
	Field 3	R^2^	0.72	0.54	0.70	0.75	0.76
		RMSE	0.153	0.185	0.155	0.146	0.136
		NRMSE %	53.18	55.60	52.20	51.86	51.50
		MAE	0.120	0.134	0.128	0.124	0.101
	Field 4	R^2^	0.87	0.55	0.85	0.88	0.89
		RMSE	0.195	0.197	0.209	0.191	0.188
		NRMSE %	31.98	33.71	31.26	30.94	30.47
		MAE	0.124	0.165	0.118	0.113	0.106

**Table 8:** RMSE and *R*^2^ values for the validation datasets using L8 and environmental data.

Sensor type	Fields	Metrics	Bands	Vegetation Indices	Band + VIs	Bands + VIs + Topographic	Band + Indices + Topographic + Climate data
Landsat 8	Field 1	R^2^	0.40	0.36	0.47	0.52	0.57
		RMSE	0.173	0.178	0.159	0.152	0.145
		NRMSE %	57.41	58.34	57.23	57.02	56.82
		MAE	0.137	0.145	0.134	0.133	0.128
	Field 2	R^2^	0.67	0.60	0.70	0.71	0.75
		RMSE	0.117	0.140	0.126	0.113	0.108
		NRMSE %	51.88	52.74	51.93	51.38	50.77
		MAE	0.135	0.145	0.132	0.128	0.113
	Field 3	R^2^	0.61	0.47	0.66	0.75	0.76
		RMSE	0.160	0.181	0.190	0.136	0.136
		NRMSE %	51.02	51.77	50.80	50.67	49.84
		MAE	0.126	0.128	0.120	0.114	0.109
	Field 4	R^2^	0.30	0.27	0.38	0.39	0.43
		RMSE	0.111	0.158	0.102	0.105	0.115
		NRMSE %	59.60	60.05	59.21	58.66	58.20
		MAE	0.149	0.158	0.150	0.147	0.143

This research was initially structured based on four key questions to explore the feasibility of PS, S2, and L8 in terms of both type and spatiotemporal resolution and how different combinations of data influence the accuracy of soybean within-field yield variability. The RF models were individually validated using a small data set and individual fields that were not used for training; this was done to ensure the sensitivity of the analysis of the results. To analyze the obtained results clearly, we generated box plots of the validation datasets (Fig. 7). In the following sections, we summarize the results of the RF analysis.

1. Which stage of soybean growth and individual satellite data image offers the most accurate estimation?

The beginning bloom growing stage (R1) had high accuracy for the estimation of soybean yield between 187 and 223 DOY as crops reached the peak vegetative periods. The availability of satellite images differed per year and location during the growing season. Considering that the frequency and available cloud-free remotely sensed imagery accuracy of crop yield prediction varies throughout the growing phase, determining a single-date satellite image is critical. The accuracy of the yield estimation models increased constantly at the beginning of July. However, July 30 and 31 gave the most accurate yield estimation results for the three satellite images. The RF model using a single image shows that soybean crop yield can be accurately estimated within the field variability at the end of July approximately 2 or 2.5 months before the harvesting period.

2. How do the spatial and temporal resolutions of PS, S2, and L8 affect the precision of yield prediction?

Coming back to question one, we observed that PS and S2 had the most promising satellite data in soybean grain yield prediction as their spatial and temporal resolutions were much finer than those of L8 (Fig. 7).

3. Does the calculation of additional VIs contribute extra information to the estimation model?

The RMSE value was almost the same for the spectral bands and VI models with slightly higher errors for the VI models alone for the training datasets. When VIs were added to the bands, the accuracy of yield estimation rose marginally but not always for the case of PS, S2, and L8 based on both training and validation models (Table 6, Table 7, Table 8; Fig. 7). The result demonstrates that the addition of VIs to the spectral bands could add some extra insight to improve the accuracy of the yield prediction.

4. How does the accuracy of estimation differ when PS, S2, and L8 spectral bands and VI datasets are combined with environmental data?

Topographic variables, including DTM, slope, and aspect, were combined first, and the model accuracy increased noticeably (Fig. 7). Further improvements were achieved by applying climate data to the prediction model (e.g., monthly rainfall and temperature).

## 4 Discussion

### 4.1 Effectiveness of RF

This research focused on how well the within-field yield variability of soybean crops could be explained using multispectral satellite images at various spatial and temporal resolutions using RF. In this study, the RF model was chosen because we discovered that the correlation between crop yield and reflectance is sophisticated enough for ML methods, which enhance within-field yield estimates. Because RF is less likely to contain outliers, it is expected to have improved yield estimation performance [43]. Additionally, the RF algorithm is effective at managing relationships that are both linear and nonlinear. The result of this study proves the effectiveness of RF regression to predict the soybean yield at the field scale with RMSE values of 0.094, 0.069, and 0.108 t/ha using PS, S2, and L8, respectively, for the validation parcels (Table 6, Table 7, Table 8). These obtained results and models were much more robust and stronger rather than those of Pejak et al. [25], who also estimated soya yield within the field level based on S2 VIs and soil data with an RMSE error of 0.553 t/ha using SGD.

### 4.2 Time series analysis of phenology

With a focus on RS for precision farming, this work was designed around four questions that cover four pertinent parameters for within-field mapping of soybean variability. First, we determined how important the temporal variations of the sensed information are, specifically the potential evaluation of phenological stages and optimal data giving accurate yield estimation through time series analysis. RS-based time series of phenological stages showed peak soybean growth in July, which took place in the Fourth node, Fifth node, and beginning bloom stages (V4–V5–R1) as this period could explain the yield variability within the field with RMSE value from 0.183 to 0.321 t/ha for the training datasets (Table 3, Table 4, Table 5; Fig. 3, Fig. 4, Fig. 5). Previous studies have revealed that seasonal peak VI values provide more accurate yield estimations [44,45]. The satellite images acquired on July 30 and 31 produced accurate yield estimations for all PS, S2, and L8 bands using RF. This result agrees with the study of Skakun et al. [29], who conducted soybean yield estimation using WorldView-3, PS, S2, and L8 satellite imagery in Iowa, USA.

### 4.3 Impact of spatial resolution on yield estimation

Second, we explored the potential capability of multispectral datasets from PS, S2, and L8 to estimate the soybean grain yield within the field variability while considering sensor variations and the trade-offs between accuracy and expense. The results showed that the high spatial resolution satellite data of PS could estimate the yield with high accuracy (RMSE = 0.114 t/ha, NRMSE = 54.2% and MAE = 0.101 t/ha), followed by S2, which had lower accuracy in terms of RMSE but higher accuracy considering the coefficient of determination (RMSE = 0.171 t/ha, NRMSE = 28.66% and MAE = 0.107 t/ha) for the test field using only basic spectral bands (Table 6, Table 7). Finally, L8 had an RMSE of 0.117 t/ha, NRMSE = 51.88% and MAE = 0.135 t/ha (Table 8). Our model findings demonstrate a decreasing yield estimation accuracy while moving from high-resolution to coarser data of 3, 10, and 30 m, respectively. From the prediction models, we could also highlight that PS bands were not always superior to S2 in explaining the soybean yield variability for some validation fields. This might have been due to the radiometric coverage being lower than that of the S2 satellite despite the high temporal and spatial resolution of PS. The lack of the SWIR bands in PS might also be a reason. Nevertheless, The opportunity to improve the predictive ability of these models and promote digital agriculture in crop modeling, forecasting, and yield estimation is provided by near-daily PS products [46]. However, many studies have described how fine spatial and temporal resolution satellite imageries (e.g., S2 and L8) often fail to solve the within-field yield variabilities that are important to performing precise agricultural applications, especially for small-scale fields (i.e., plots smaller than 2 ha) [47]. For instance, L8 images can contain different spectral information because of the coarse 30-m spatial resolution.

Third, VIs derived from each satellite image, added to the model as extra information, were analyzed. Previous studies developed empirical connections between crop yield and VIs or biophysical factors (such as the LAI) to estimate the yield in large homogenous crop plots [25,48]. In this research, the use of VIs and basic spectral bands together demonstrated improved accuracies for all PS, S2, and L8 data, but not all the time. However, some studies found that calculating separate VIs could not improve yield accuracy estimations [42]. This would mean that RF can derive from individual satellite bands themselves pertinent data for yield estimation that are often supplied by VIs.

Fourth, we evaluated the effect of environmental datasets combined with the basic spectral bands with VIs in regression analysis. A combination of environmental data with PS, S2, and L8 data provided the highest and most accurate soybean yield estimation and outperformed previously established models. Numerous research has combined environmental data with satellite data to support crop yield estimation, frequently using crop simulation models [19,49]. The integration of environmental data with PS showed the most accurate yield estimation for the training datasets (RMSE = 0.165 t/ha, NRMSE = 28.95% and MAE = 0.098 t/ha) (Fig. 3). In this study, we used two kinds of static and changeable environmental data for the analysis. The first one is topographic, which is constant throughout the growing season, whereas the second one comprises unstable climate variables.

However, this study has some limitations which might affect the model performances that need to be considered. The used climate data had a coarse pixel size of 4 km, and higher spatial resolution data would increase the accuracy further and detect the precipitation and temperature variation within the study site. However, finer pixel-size meteorological data for the study site were not available. Besides, this research considered only ground-truth data at least a little from the GPS combined tractor. This may cause a problem when applying the methodology to other regions where such modern combine harvesters are not used, especially, in developing countries. The aforementioned factors can affect the accuracy and reproducibility of the model in other countries.

Finally, we talked about the scope of the findings related to precision farming provided here.

## 5 Conclusions

This article compared the performance of the high and coarse spatiotemporal resolutions of the satellite imagery of S2 and L8 in soybean yield estimation within the field variability with *R*^2^ ranging from 0.55 to 0.71 for 3-m PS, from 0.7 to 0.88 for 10-m S2, and from 0.38 to 0.7 for L8 data (RMSE of 0.111, 0.076, and 0.126 t/ha, respectively) with the RF ML algorithm. The introduction of environmental datasets (topographic and climatic) to the basic PS, S2, and L8 data provided further improvements and an accurate yield estimation model within the soybean yield variability, with *R*^2^ that varied from 0.7 to 0.87 for PS, 0.73 to 0.90 for S2, and 0.43 to 0.76 for L8. To the best of our knowledge, no studies have yet used both topographical and climate variables together with satellite images for high-resolution soybean yield mapping. Meanwhile, only a few studies focused on using weather data combined with satellite-based VIs. Furthermore, this is the first case study that uses eight bands of new PS imagery for soybean yield prediction at the field level. Only a scarce number of studies have assessed multisource satellite data on within-field soybean yield. In consideration of these implications for precision agriculture, this study offers new methodological breakthroughs in within-field soybean yield estimation when comparing the time series of phenological stages from all three sensors. We found that crops reached their maximum growth in July (V4–V5–R1 growing stages) and provided higher yield estimation. The optimal date to predict the soybean yield within the field scale was approximately 60 or 70 days before harvesting periods during the beginning bloom stage. This developed model can be applied for other crops and locations when suitable training yield data are available. Further studies should focus on deep learning algorithms for crop yield forecasting with hyperspectral and synthetic aperture radar.

## Author contribution statement

Khilola Amankulova-conceived and designed the experiments; performed the experiments; analyzed and interpreted the data; wrote the paper.

Nizom Farmonov-performed the experiments; contributed reagents, materials, analysis tools or data; wrote the paper.

Parvina Akramova-contributed Conceived and designed the experiments; analyzed and interpreted the data; wrote the paper.

Ikrom Tursunov-Performed the experiments. Analyzed and interpreted the data. Contributed reagents, materials, analysis tools or data.

László Mucsi-contributed reagents, materials, analysis tools or data; conceived and designed the experiments.

## Data availability statement

Data will be made available on request.

## Additional information

Supplementary content related to this article has been publish online at [URL].

## Declaration of competing interest

The authors declare that they have no known competing financial interests or personal relationships that could have appeared to influence the work reported in this paper
