# Supplementary material for: Comparison of PlanetScope, Sentinel-2, and landsat 8 data in soybean yield estimation within-field variability with random forest regression
Source: Heliyon. 2023 Jun 19;9(6):e17432. doi: 10.1016/j.heliyon.2023.e17432 (PMC10319221; doi:10.1016/j.heliyon.2023.e17432)
Supplement: Multimedia component 1 [file mmc1.pdf]

<u>Form to confirm authorship changes for *Heliyon*</u>

This form must be ***signed by all authors*** when there is a change in authorship which includes changes to any of the following items: author name(s), order of the authors, the corresponding author(s), the addition of authors, the removal of authors and changes in affiliation.

By personally signing this note, **all** authors confirm that: I) the changes are in accordance with their scientific contribution, II) they agree with all the changes and III) confirm that the authorship list conforms to the authorship criteria outlined on [*Heliyon*'s ethics page](). IV) it is the responsibility of the corresponding author to get the signature from all co-authors accepting the change. In case of any ethic violation/malpractice in the signature, the corresponding author is accountable. The completed form should be returned along with the final/revised manuscript to proceed further with the manuscript. Manuscripts for which incomplete forms have been submitted will be rejected within 5 working days.

Any disputes on the authorship list and contributions need to be resolved by the involved scientists and *Heliyon* will only proceed with the evaluation of the manuscript once we receive confirmation, through this form, that such an agreement between the authors has been reached.

Manuscript number: HELIYON-D-22-33618R1

Article title: Comparison of PlanetScope, Sentinel-2, and Landsat 8 data in soybean yield estimation within field variability with Random Forest Regression

Complete new author list: Khilola Amankulova, Nizom Farmonov, Parvina Akramova, Ikrom Tursunov, László Mucsi

Date: 09.06.2023

#	First name	Last name	Order change (Y/N)	Addition / Deletion	Change in Author name (Y/N)	Affiliation Change (Y/N)	Reason for the change	Signature
1	Khilola	Amankulova	N		N	N		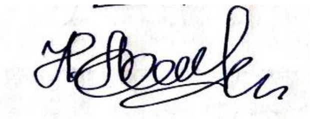

2	Nizom	Farmonov	N		N	N		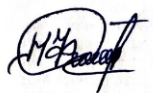
3	Parvina	Akramova		Addition			During the revision processes, she contributed significantly to the following sections:<br>Conceived and designed the experiments.<br>Analyzed and interpreted the data.<br>Wrote the paper	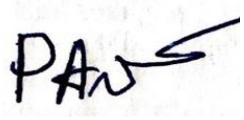
4	Ikrom	Tursunov		Addition			During the revision processes, he contributed significantly to the following sections:<br>Performed the experiments.<br>Analyzed and interpreted the data.<br>Contributed reagents, materials, analysis tools or data;	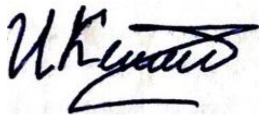
5	László	Mucsi	Y				As supervisor of all researchers, I must be at the end of the author list.	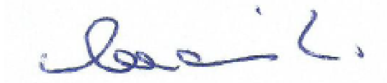
